# Combining LSD1 and JAK-STAT inhibition targets Down syndrome-associated myeloid leukemia at its core

**DOI:** 10.1038/s41375-022-01603-3

**Published:** 2022-05-24

**Authors:** Juliane Grimm, Raj Bhayadia, Lucie Gack, Dirk Heckl, Jan-Henning Klusmann

**Affiliations:** 1 Pediatric Hematology and Oncology, Martin Luther University Halle-Wittenberg, Halle, Germany; 2 Department of Internal Medicine IV, Oncology/Hematology, Martin Luther University Halle-Wittenberg, Halle, Germany; 3 Department of Pediatrics, Goethe University Frankfurt, Frankfurt am Main, Germany; 4 Frankfurt Cancer Institute, Goethe University Frankfurt, Frankfurt am Main, Germany; 5 German Cancer Consortium (DKTK), Partner Site Frankfurt/Mainz, and German Cancer Research Center (DKFZ), Heidelberg, Germany

**Keywords:** Acute myeloid leukaemia, Targeted therapies

Individuals with Down syndrome (DS) are predisposed to developing acute megakaryoblastic leukemia (ML-DS) within their first years of life [1]. Although, ML-DS is associated with a favorable prognosis, children with DS often experience severe toxicities from chemotherapy [2]. This highlights the unmet need for targeted therapies with improved risk profiles in this entity.

Consequently, the aim of this study was to investigate a novel therapeutic approach specifically tailored to intervene with hallmarks of ML-DS leukemogenesis. The evolution of ML-DS occurs in a step-wise process originating from pre-malignant transient abnormal myelopoiesis (TAM) [3]. The molecular mechanisms underlying the progression from TAM to ML-DS are not fully understood. However, it was previously shown that epigenetic changes play a pivotal role in ML-DS leukemogenesis. The lysine demethylase LSD1 was identified as a crucial player in this process, as LSD1-driven gene signatures become activated in ML-DS [4]. Accordingly, RNA-sequencing analysis of pediatric acute myeloid leukemia (AML) subtypes revealed that LSD1 was highly expressed in acute megakaryoblastic leukemia (AMKL), and especially in TAM and ML-DS patients (Supplementary Fig. 1). LSD1 is essential for hematopoiesis, particularly during granulocytic and erythroid differentiation [5], and was shown to contribute to differentiation blockade in different AML subtypes [6–8]. Consequently, various irreversible LSD1 inhibitors have been developed, with some currently undergoing clinical trials for AML [9]. Therefore, we sought to investigate the rational use of LSD1 inhibitors in pediatric AMKL. The non-DS-AMKL cell line M-07e and the ML-DS cell line CMK were highly sensitive to irreversible LSD1 inhibition (IC50_M-07e_ = 9.1 nM; IC50_CMK_ = 38.8 nM; Supplementary Fig. 2A). Testing serial dilutions of the irreversible LSD1 inhibitor in non-DS-AMKL and ML-DS patient samples expanded via xenotransplantation (see Supplementary Table 1 for patient characteristics), both entities were equally sensitive to LSD1 inhibition (non-DS-AMKL: IC50#1 = 15.0 nM, IC50#2 = 2.0 nM; ML-DS: IC50#1 = 31.2 nM, IC50#2 = 17.1 nM, IC50#3 = 3.8 nM). All dose-response curves plateaued at a certain LSD1 inhibitor concentration (Supplementary Fig. 2B). The non-linear relationship between cytotoxicity and dosage points toward proliferation arrest and differentiation in response to LSD1 inhibition. In line with this, we observed myeloid differentiation upon visual inspection (Supplementary Fig. 3A) and upregulation of the myeloid markers CD86 and CD11b after 3 days of LSD1 inhibitor treatment (Supplementary Fig. 3B).

These results revealed a potent proliferation block and induction of differentiation in non-DS-AMKL and ML-DS samples, however, the therapeutic efficacy of LSD1 inhibition may be limited by its non-linear dose-response relationship. Consequently, we aimed to design a rational drug combination to increase its anti-leukemic effects. Another hallmark of ML-DS development is the acquisition of activating mutations in Janus kinases (JAK) and cytokine receptors [4], promising potent anti-leukemic effects of the combination of LSD1 inhibition and the JAK1/JAK2 inhibitor ruxolitinib, as it was previously proposed for *JAK2*^*V617F*^ mutated myeloproliferative neoplasms, secondary AML and a *CSF3R*^mut^/*CEBPα*^mut^ AML model [10–12]. Accordingly, pre-treatment with 350 nM LSD1 inhibitor for 3 days followed by exposure to serial dilutions of ruxolitinib led to synergistic growth inhibition in non-DS-AMKL and ML-DS cell lines (Supplementary Fig. 4), as well as in all ML-DS patient samples (Fig. 1A). The combination of LSD1 inhibition and ruxolitinib proved to be very effective in non-DS-AMKL blasts, however, with only additive cytotoxic effects in one of the two patient samples (Fig. 1A). Drug synergy in the ML-DS samples was confirmed when calculating the Bliss synergy scores (Fig. 1B). Interestingly, samples ML-DS #1 (*JAK1*^mut^) and #2 (wild-type for *JAK1*, *JAK2*, and *JAK3*, Supplementary Fig. 5) showed particularly high synergy scores (ML-DS #1 synergy score = 10.4; ML-DS #2 synergy score = 15.6; Fig. 1B). Contrary, the *JAK3*^mut^ patient sample ML-DS #3 (Supplementary Fig. 5) only displayed mild drug synergy between LSD1 inhibition and ruxolitinib (synergy score = 2.0; Fig. 1B). Consequently, as ruxolitinib is a JAK1/JAK2 inhibitor, synergistic anti-leukemic effects seem to depend on *JAK* mutational status, which must be considered in future pre-clinical and clinical testing of this drug combination for ML-DS patients.

**Fig. 1:**
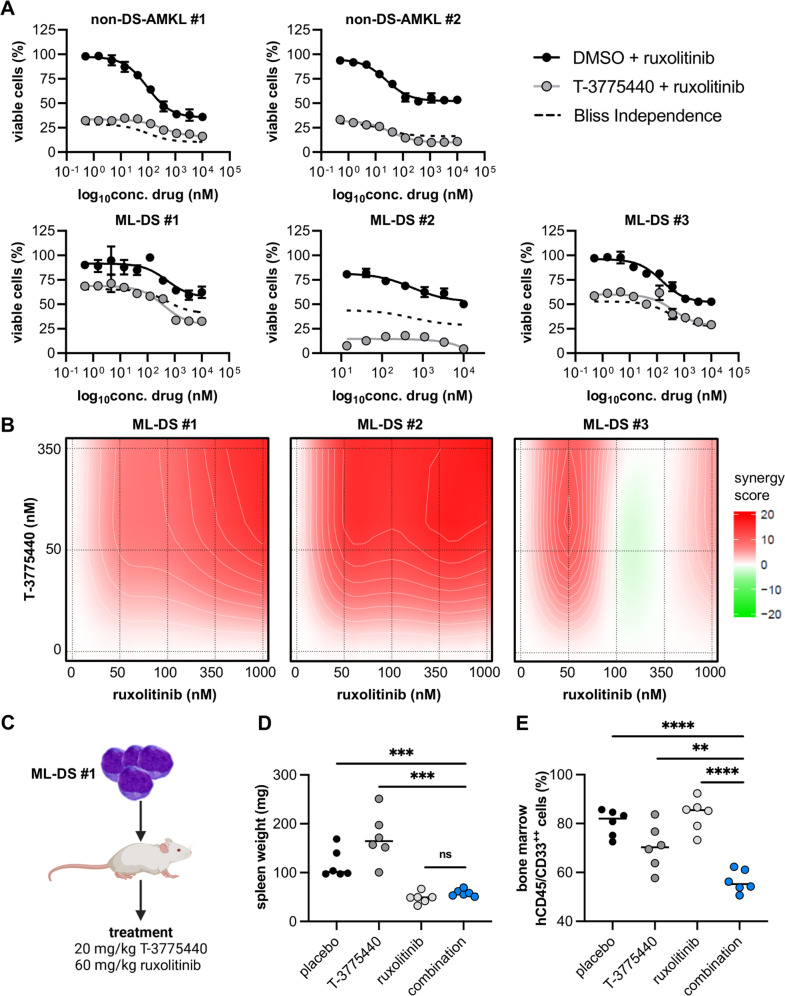
Combined LSD1 inhibition and JAK-STAT signaling blockade synergize to produce anti-leukemic effects in ML-DS. **A** Dose-response curves depicting cell viability of different ML-DS and non-DS-AMKL patient samples (expanded via xenotransplantation) after treatment with DMSO or 350 nM T-3775440 for 6 days, with the addition of serial dilutions of ruxolitinib from day 3 to day 6. All cell viability values were normalized to the corresponding all DMSO control. **B** Synergy blots displaying the color-coded Bliss synergy score, calculated after treatment of different ML-DS patient samples (expanded via xenotransplantation) with T-3775440 and ruxolitinib for 6 days. Interpretation of synergy scores: >10 drug synergy; −10 to 10 additive effects; <−10 drug antagonism. **C** Treatment schedule of humanized recipient mice transplanted with the ML-DS #1 patient sample. **D** Spleen weights in milligrams of mice engrafted with the ML-DS #1 patient sample, after treatment with placebo, T-3775440, ruxolitinib, or the combination of both drugs for 7 days. **E** Bone marrow infiltration in mice engrafted with the ML-DS #1 patient sample, after treatment with placebo, T-3775440, ruxolitinib, or the combination of both drugs for 7 days. Infiltration was defined as percentage of human myeloid blasts and was measured by flow cytometry. Human myeloid blasts were defined as CD45^+^ and CD33^+^. ns *p* > 0.05, **p < 0.01, ***p < 0.001, ****p < 0.0001; *p* values are derived from two-tailed Student’s *t* tests comparing two groups. ML-DS myeloid leukemia associated with Down syndrome, non-DS-AMKL acute megakaryoblastic leukemia not associated with Down syndrome, DMSO dimethyl sulfoxide.

Further corroborating the observed drug synergy, we demonstrated only limited apoptosis rates when ruxolitinib or the LSD1 inhibitor were used as monotherapies, whereas the combination synergistically increased the percentage of apoptotic cells in ML-DS samples and in one out of two non-DS-AMKL samples (Supplementary Fig. 6). Moreover, LSD1 inhibition alone or in combination with ruxolitinib effectively blocked G1 to S phase transition (Supplementary Fig. 7). This effect was particularly strong in the ML-DS sample.

To further investigate the synergistic anti-leukemic effects of LSD1 inhibition and JAK-STAT blockage in vivo, we treated recipient mice with stable engraftment (median peripheral blasts 3.0%, Supplementary Fig. 8) of ML-DS blasts (*JAK1*^mut^; Fig. 1C). At the end of the treatment period, we observed significantly reduced spleen weight in mice treated with ruxolitinib (median 49.2 mg) and the combination therapy (median 56.8 mg) as opposed to the placebo (median 101.2 mg) and LSD1 inhibitor (median 157.0 mg) group (Fig. 1D). The spleen infiltration by myeloid blasts was reduced in both monotherapies and the drug combination compared to the placebo group (median percentage of human CD45^+^/CD33^+^ cells: placebo—28.4%, LSD1 inhibitor—4.8%, ruxolitinib—3.9%, combination therapy—4.2%, Supplementary Fig. 9). Of note, the combination of the LSD1 inhibitor and ruxolitinib was the only treatment regimen that achieved significant reduction of leukemic burden in the bone marrow (median percentage of human CD45^+^/CD33^+^ cells: placebo—82.0%, LSD1 inhibitor—69.5%, ruxolitinib—85.5%, combination therapy—55.2%, Fig. 1E), underlining the synergistic cytotoxic effect of both drugs in ML-DS.

To unravel the molecular mechanisms behind the synergy between LSD1 inhibition and disruption of JAK-STAT signaling, we performed RNA-sequencing of the patient samples ML-DS #1 and #2 after treatment with DMSO, LSD1 inhibitor, ruxolitinib, or the combination of both drugs. Gene expression was normalized to vehicle control, identifying 552 differentially expressed genes (*p* < 0.05). The gene expression signature in the combination therapy was mainly driven by LSD1 inhibition, since only one gene was significantly divergently expressed between the LSD1 inhibitor and the combination samples (*SLCO2B1*, Fig. 2A). We uncovered four different gene expression clusters, with two clusters displaying cooperative inhibition (cluster 1) and induction (cluster 3) of gene expression by LSD1 inhibition and ruxolitinib (Fig. 2A). Gene ontology analyses of the clusters revealed that LSD1 inhibition and ruxolitinib repressed hallmarks of cell division, as genes essential for cell cycle checkpoints, synthesis of DNA, and fatty acyl-CoA biosynthesis were downregulated in cluster 1 (Fig. 2B). Of note, genes involved in the transition from G1 to S phase were also repressed by the combination therapy, in line with the results from our BrdU assay (Supplementary Fig. 7). Independently from ruxolitinib, the LSD1 inhibitor downregulated a plethora of genes involved in DNA replication (cluster 2, Fig. 2B). Adding to the anti-proliferative gene signature, ruxolitinib and LSD1 inhibitor cooperatively elevated the expression of negative regulators of DNA transcription (cluster 3, Fig. 2B). In our in vitro studies, we observed that LSD1 inhibition induces myeloid differentiation—a finding that was reflected by the upregulation of genes involved in neutrophil degranulation (cluster 4, Fig. 2B). We also demonstrated upregulation of immunological gene expression patterns, e.g., MHC-II antigen presentation, and interferon gamma signaling (cluster 4, Fig. 2B), upon LSD1 inhibition as monotherapy or in combination with ruxolitinib. This is in accordance with previous studies in solid tumors, where LSD1 inhibition has been shown to increase anti-tumor T cell immunity [13]. Additionally, the expression of gene signatures involved in activation of cytokine signaling was driven by LSD1 inhibition (cluster 4, Fig. 2B). Elevated cytokine signaling and the enrichment of differentiation pathways were validated by gene set enrichment analysis on the entire set of differentially expressed genes (Supplementary Fig. 10).

**Fig. 2:**
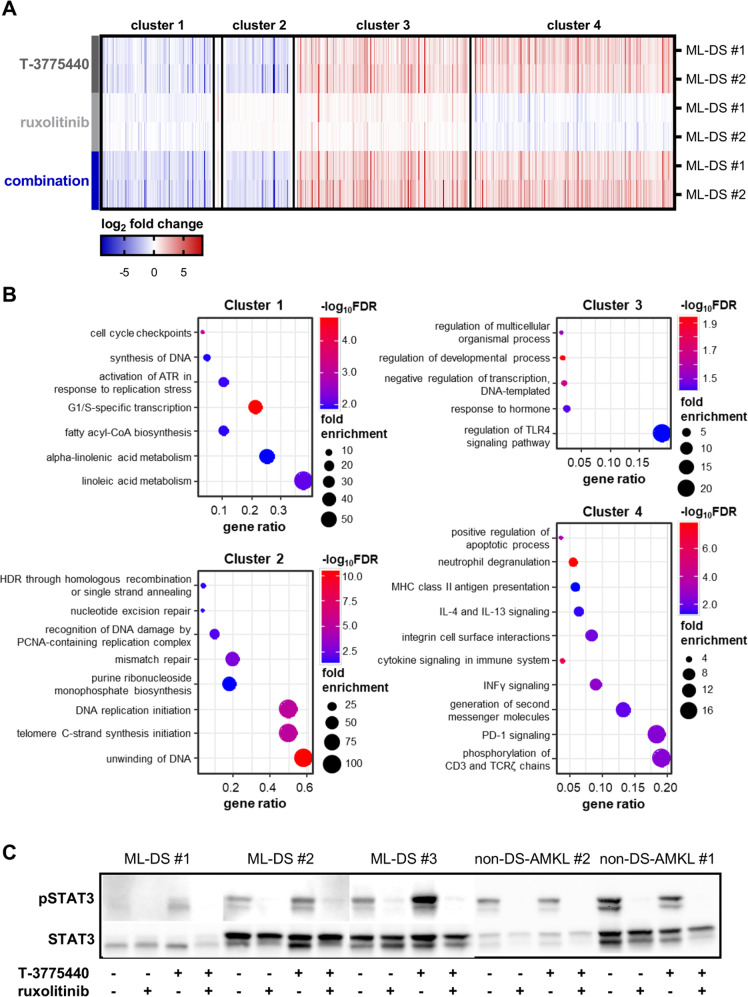
Transcriptomic profiling reveals gene expression signature driven by LSD1 inhibition with induction of cytokine signaling. **A** Heatmap of the 552 differentially expressed genes across all treatment groups (T-3775440, ruxolitinib, combination). Two ML-DS patient samples were used in all groups. Gene expression is normalized to the respective DMSO control and depicted as color-coded log_2_ fold-change. **B** Gene ontology analysis of the four identified gene clusters from **A**. The gene ratio was defined as the number of identified genes in a certain biological process, normalized to the total number of genes belonging to this biological process. **C** Western blot of phosphorylated STAT3 and total STAT3 in ML-DS and non-DS-AMKL patient samples (expanded via xenotransplantation) after 3 days of treatment with DMSO, T-3775440, ruxolitinib, or the combination of both drugs. ML-DS myeloid leukemia associated with Down syndrome, DMSO dimethyl sulfoxide, non-DS-AMKL acute megakaryoblastic leukemia not associated with Down syndrome, FDR false discovery rate.

In our study, the induction of cytokine signaling was limited to the ML-DS context, since we observed increased STAT3 phosphorylation after LSD1 inhibitor treatment in all ML-DS but not in the non-DS-AMKL patient samples (Fig. 2C). The addition of ruxolitinib after LSD1 pre-treatment completely abrogated STAT3 signaling in all tested patient samples (Fig. 2C). These results suggest that activation of cytokine signaling might be an important component of the drug synergy between LSD1 inhibition and blockage of the JAK-STAT pathways. ML-DS blasts thrive on aberrant JAK-STAT signaling and LSD1 inhibition could cause an even greater dependence on constitutively active JAK-STAT pathways—a stimulus which is then abruptly revoked by ruxolitinib.

Taken together, we are the first to demonstrate synergistic anti-leukemic effects using the combination of an irreversible LSD1 inhibitor and the JAK1/JAK2 inhibitor ruxolitinib in ML-DS, with both drugs being specifically selected to target core molecular features of the leukemic transformation from TAM to ML-DS [4]. Although, consistent drug synergy was only observed in ML-DS, the combination of LSD1 inhibition and JAK blockage still seems to be a promising therapeutic approach in non-DS-AMKL. Overall, this opens the avenue for clinical concepts combining LSD1 and JAK inhibitors, tailored to the patients’ JAK mutational status assuring optimal synergistic efficacy.

## Supplementary information

Supplemental Material
