# Supplementary material for: Combining LSD1 and JAK-STAT inhibition targets Down syndrome-associated myeloid leukemia at its core
Source: Leukemia. 2022 May 24;36(7):1926–30. doi: 10.1038/s41375-022-01603-3 (PMC9252908; doi:10.1038/s41375-022-01603-3)
Supplement: Supplementary file 1 — Supplemental Material [file 41375_2022_1603_MOESM1_ESM.pdf]

Supplemental Material

Combining LSD1 and JAK-STAT inhibition targets Down syndrome-associated myeloid leukemia at its core

Grimm et al.

Supplemental Methods

Cell culture and patient samples

All human cell lines were purchased from the German National Resource Center for Biological Material (DSMZ, Braunschweig, Germany) and cultured as recommended by the supplier. Patient samples (expanded via xenotransplantation) were cultured in StemSpan (StemCell Technologies, Vancouver, Canada) supplemented with streptomycin/penicillin (Thermo Fisher Scientific, Waltham, MA, USA) and a cytokine cocktail (FLT3-L, SCF, IL-6, IL-3, TPO; all purchased from PeproTech, Rocky Hill, NJ, USA). Samples were taken from pediatric AML patients treated as part of the AML Berlin-Frankfurt-Münster study group. Written informed consent was given by all custodians of the participants in consent with the Declaration of Helsinki and the study was approved by the local ethic committees of the participating institutions.

Evaluation of drug response and synergy analyses

Cell lines and patient samples were treated for six days. Experiments were carried out in at least three independent replicates for cell lines and at least two independent replicates for primary material. On day 0 and day 3, a serial dilution of T-3775440 (Hycultec, Beutelsbach, Germany) was added to the cells. Medium was supplemented with fresh ruxolitinib (Hycultec) from day 3 to 5. Cell viability was determined on day 6 using the CellTiter-Glo® 2.0 Cell Viability Assay (Promega, Madison, WI, USA) according to the manufacturer's recommendations. Synergy was analyzed using the SynergyFinder web application (version 2.0).[1]

Flow cytometry: differentiation, apoptosis and cell cycle assays

All flow cytometry analyses were performed using the CytoFLEX platform (Beckman Coulter, Brea, CA, USA). For assessment of myeloid differentiation after T-3775440 treatment, anti-CD11b-PC7 (Beckman Coulter) and anti-CD86-Alexa Fluor® 700 (BD Biosciences, Franklin Lakes, NJ, USA) were applied. Evaluation of apoptosis and cell cycle progression was performed using the Annexin V Apoptosis Detection Kit II and the BrdU Flow Kit (BD Biosciences), respectively. Both assays were performed according to manufacturer's recommendations.

In vivo experiments

Humanized immunodeficient mice[2] were bred and maintained at the animal facility of the Martin-Luther-University Halle-Wittenberg. Patient samples (expanded via xenotransplantation) were transplanted via tail vein into sublethally irradiated female mice (n=6 mice per treatment group) aged 8 to 16 weeks. For monitoring of peripheral engraftment, mice were bled every two weeks starting from six weeks after transplantation. Upon stable

engraftment, treatment with T-3775440 (20 mg/kg) and/or ruxolitinib (60 mg/kg) was initiated. Both drugs were administered once daily via oral gavage. After a treatment period of 7 days the experiment was terminated. Leukemic infiltration of the spleen and bone marrow was evaluated by flow cytometry applying a standard antibody panel consisting of anti-mouse CD45-FITC, anti-human CD45-APC, anti-human CD33-PE, anti-human CD3-APC-H7, and anti-human CD34-PerCP-Cy™5.5 (all purchased from BD).
All *in vivo* experiments were approved by local authorities and were performed in accordance with national laws and regulations. Sample size was calculated performing a power analysis using G*Power 3.1.[3]

mRNA sequencing

For total mRNA sequencing analysis, two ML-DS samples (expanded via xenotransplantation) were treated with DMSO, T-3775440, ruxolitinib or the combination of T-3775440 and ruxolitinib for two days. Afterwards, cells were harvested and RNA was isolated using the Quick RNA Miniprep kit (Zymo Research, Irvine, CA, USA) following the supplied protocol. mRNA sequencing, alignment of the RNA reads to GRCh38 genome, and calculation of differentially expressed genes were performed by Novogene (Cambridge, UK). The clustering was performed based on concordant and opposed gene regulation signatures in the monotherapies. Gene set enrichment analysis (GSEA) and gene ontology analysis using the PANTHER classification system were performed as previously described.[4–7] The mRNA sequencing data was deposited in the Gene Expression Omnibus (GEO, accession number GSE201093).

Western Blotting

Cells were harvested after treatment with DMSO, T-3775440, ruxolitinib or both drugs in combination and lysed using RIPA buffer (Thermo Fisher Scientific). Protein lysates were separated by SDS-PAGE and blotted onto a PVDF membrane (BioRad, Hercules, CA, USA). Staining was performed using the following antibodies: anti-human STAT3, anti-human phosphor-STAT3 (Y705) (Cell Signaling Technology, Danvers, MA, USA). Imaging was performed using the ChemiDoc Imaging System (BioRad).

Statistical analyses

For statistical analyses, Graphpad PRISM software (version 9.0.1) and R-Studio (R version 3.6.3)[8] were used. Two-tailed Student's t-tests were used for comparisons between two groups. $P<0.05$ was considered statistically significant. All statistical tests and sample numbers are disclosed in the respective figure legends.

Supplemental Tables

Supplemental Table 1. Clinical characteristics of patient samples used in this study.

	gender	age at diagnosis (months)	WBC (10^9/L)	hemoglobin (g/dl)	bone marrow blasts (%)	CNS involvement	allogeneic stem cell transplantation	molecular genetics	cytogenetics	response	relapse
ML-DS #1	female	13	168.0	7.1	16.0	no	no	*NRAS*^{mut}	NA	CCR	no
ML-DS #2	male	16	4.8	12.0	10.5	no	no	*GATA1*^{mut}	NA	CCR	no
ML-DS #3	male	26	32.5	7.9	70.5	no	yes	*GATA1*^{mut}	47,XY,t(3;13)(q?26;q?13~14)del(13)(q?14q22),+21c[cp14]/47,sl,del?(15)(q?)[cp2]/46,XY[1]	NR, CCR after allogeneic SCT	no
non-DS-AMKL #1	male	12	40.0	9.9	64.0	no	no	*KMT2A*^{mut}	46,XY[15].nuc ish 3q26(EVI1x2)[100/100],8q22(RUNX1T1x2),21q22(RUNX1x2)[98/100],11q23(MLLx2)[99/100], 16q22(CBFBx2)[100/100] 17q21.1(RARAx2)[100/100]	CCR	no
non-DS-AMKL #2	male	2	33.8	9.5	34.0	yes	yes	NA	46,XY,t(1;8;22)(p13;q22;q13)[14]/46,XY[1]	NR	yes

Supplemental Figures

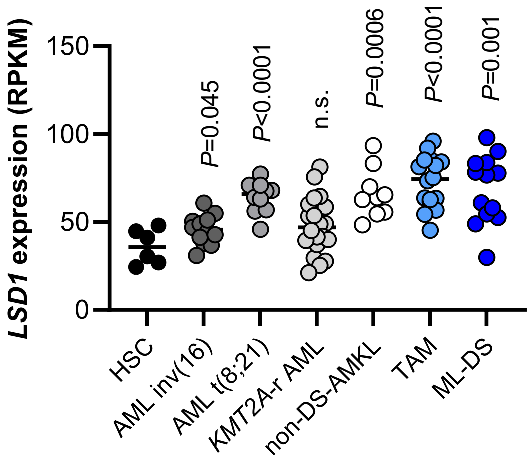

Supplemental Figure 1. LSD1 is highly expressed in pediatric AMKL. LSD1 expression in different AML subgroups displayed as RPKM (RNA-seq). The *P* values result from pairwise comparisons with normal HSC.

AMKL, acute megakaryoblastic leukemia; AML, acute myeloid leukemia; RPKM, reads per kilobase of transcript per million mapped reads; HSC, hematopoietic stem cells.

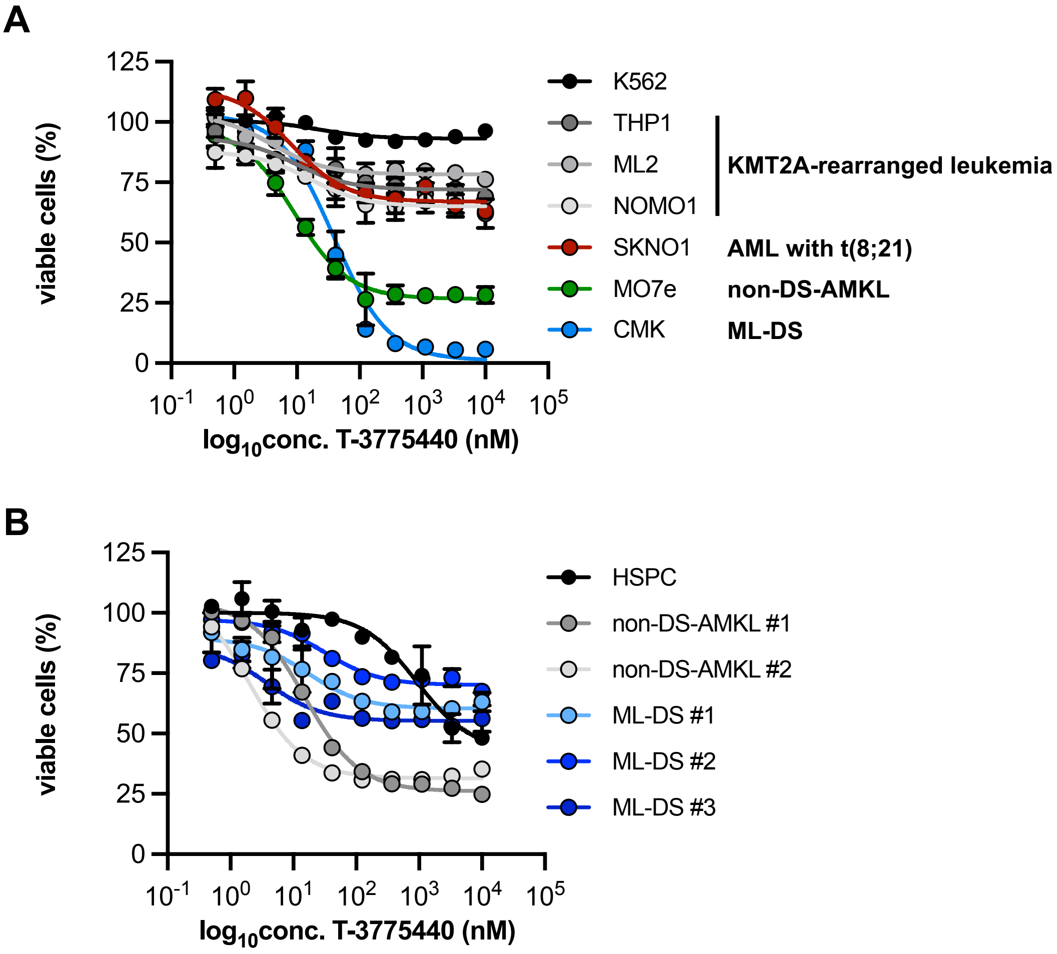

Supplemental Figure 2. ML-DS and non-DS-AMKL cells are highly sensitive to LSD1 inhibition. Dose-response curves depicting cell viability of (A) different pediatric AML cell lines and (B) CD34$^+$ HSPC and patient samples (expanded via xenotransplantation) after treatment with serial dilutions of the LSD1 inhibitor T-3775440 for six days. All cell viability values were normalized to the corresponding DMSO control.

ML-DS, myeloid leukemia associated with Down syndrome; non-DS-AMKL, acute megakaryoblastic leukemia not associated with Down syndrome; AML, acute myeloid leukemia; HSPC, hematopoietic stem and progenitor cells; DMSO, dimethyl sulfoxide.

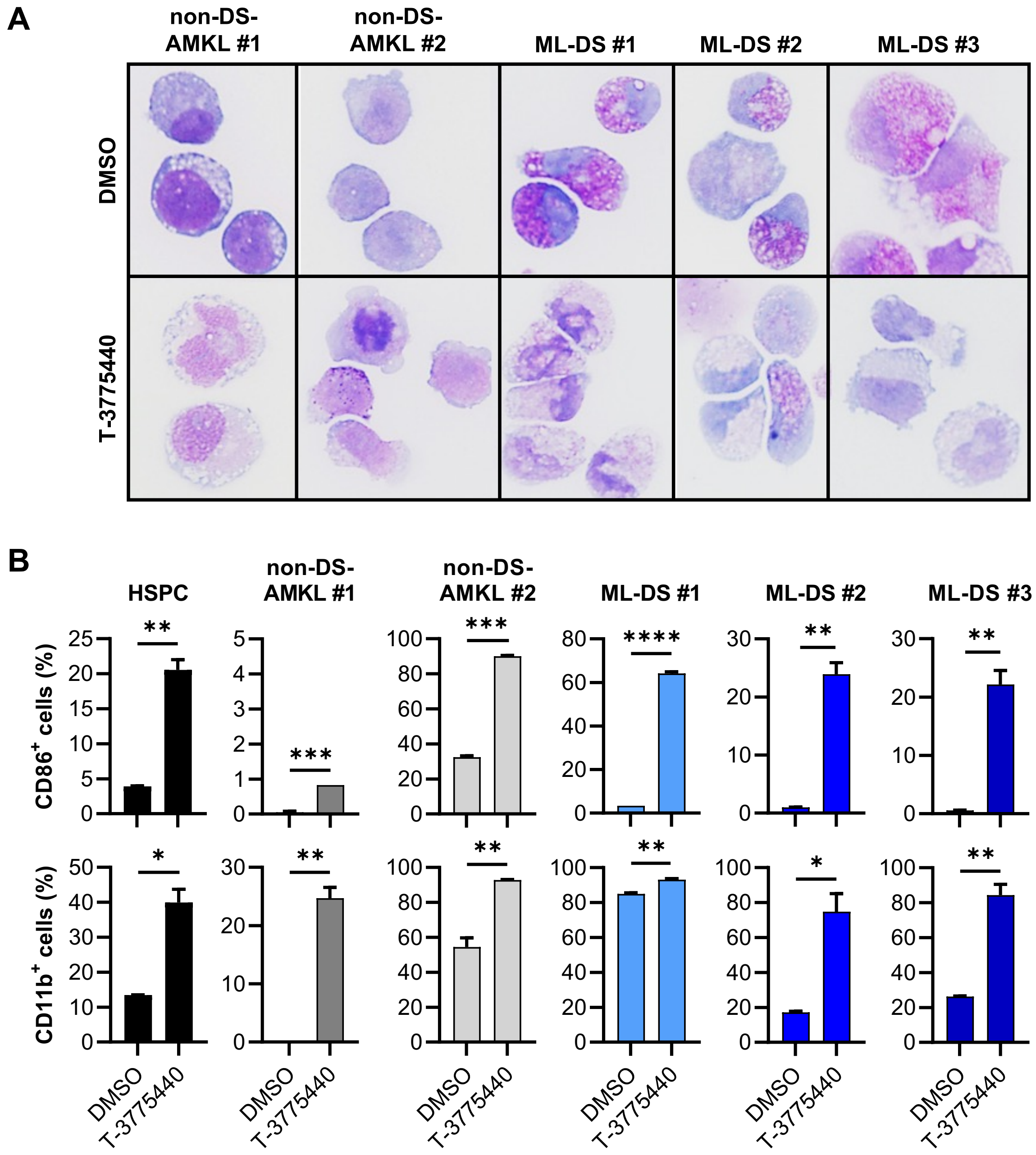

Supplemental Figure 3. Pharmacological LSD1 inhibition potently induces myeloid differentiation in ML-DS and non-DS-AMKL cells. (A) May–Grünwald–Giemsa stained cytospins of patient blasts treated with DMSO or 350nM T-3775440 for three days. (B) Bar plots depicting cell surface expression of CD86 or CD11b on HSPC and patient blasts after treatment with DMSO or T-3775440 for three days. Surface marker expression was determined via flow cytometry. **P*<0.05, ***P*<0.01, ****P*<0.001, *****P*<0.0001; *P* values are derived from two-tailed Student's t-tests comparing two groups.

ML-DS, myeloid leukemia associated with Down syndrome; non-DS-AMKL, acute megakaryoblastic leukemia not associated with Down syndrome; HSPC, hematopoietic stem and progenitor cells; DMSO, dimethyl sulfoxide.

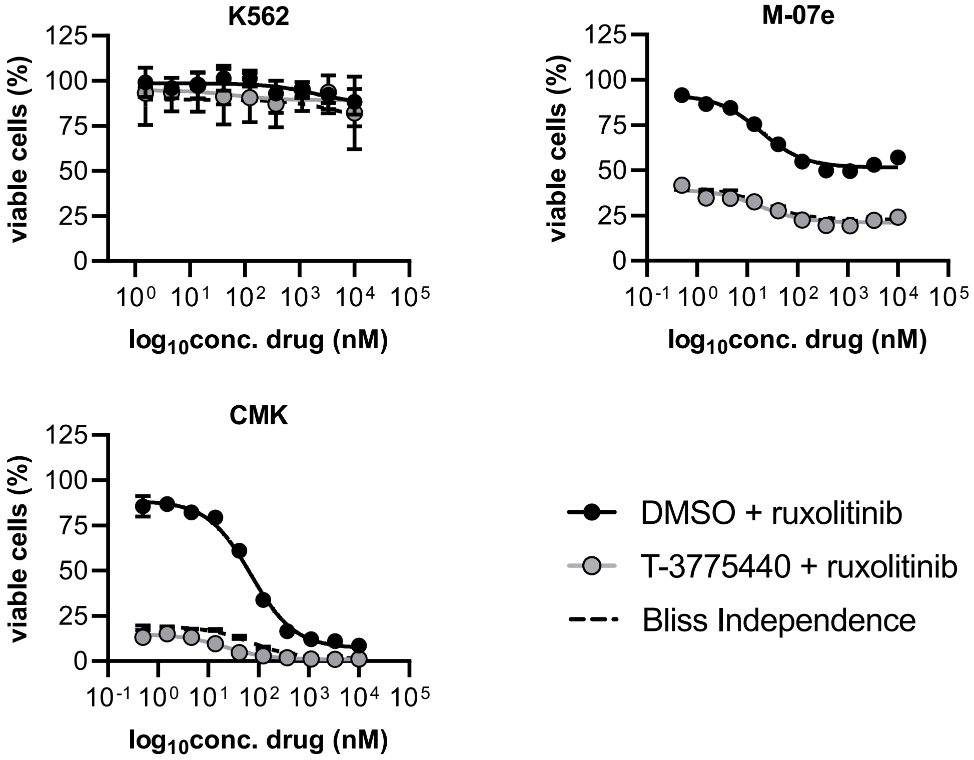

Supplemental Figure 4. The combination of the LSD1 inhibitor T-3775440 and ruxolitinib exerts synergistic cytotoxic effects in the non-DS-AMKL cell line M-07e and the ML-DS cell line CMK. Dose-response curves depicting cell viability of different AML cell lines after treatment with DMSO or 350nM of T-3775440 for six days and the addition of serial dilutions of ruxolitinib from day 3 to day 5. All cell viability values were normalized to the corresponding all DMSO control.

Non-DS-AMKL, acute megakaryoblastic leukemia not associated with Down syndrome; ML-DS, myeloid leukemia associated with Down syndrome; AML, acute myeloid leukemia; DMSO, dimethyl sulfoxide.

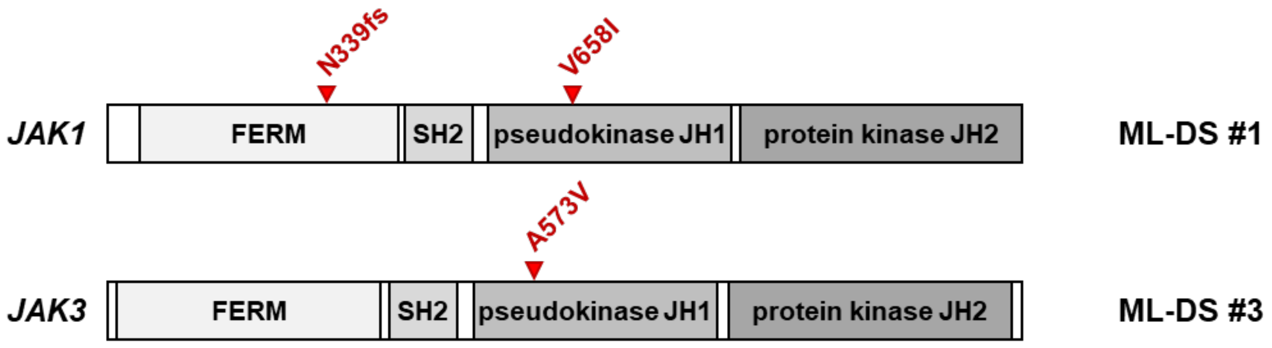

Supplemental Figure 5. Schematic of the Janus kinases 1 and 3. Depiction of the different Janus kinase domains and the locations of mutations identified in the ML-DS #1 and #3 patient samples. Adapted from Labuhn et al.

ML-DS, myeloid leukemia associated with Down syndrome.

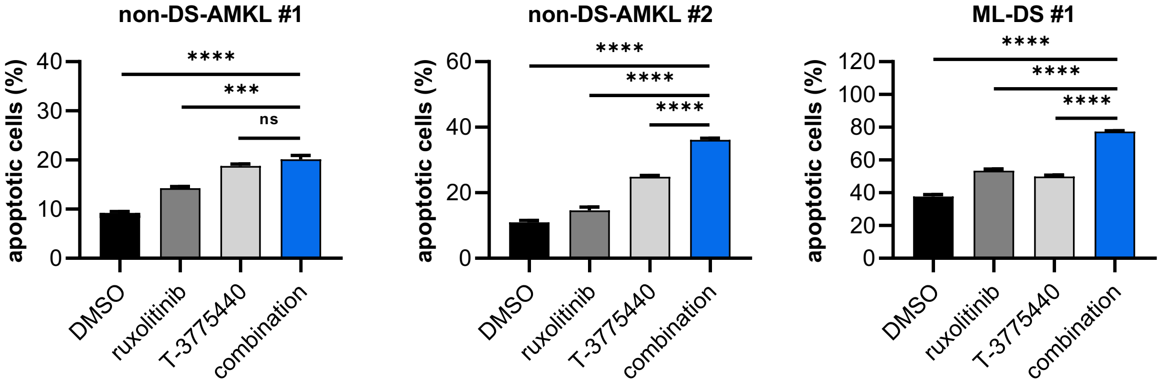

Supplemental Figure 6. The combination of LSD1 inhibition and ruxolitinib synergistically induces apoptosis. Bar plots depicting percentage of apoptotic cells after treating patient samples (expanded via xenotransplantation) with DMSO, 1µM ruxolitinib, 350nM T-3775440, or the combination of T-3775440 and ruxolitinib for three days. Apoptosis was determined using flow cytometry on DAPI and Annexin V. All Annexin V positive cells were considered apoptotic. ns P>0.05, ***P<0.001, ****P<0.0001; P values are derived from two-tailed Student's t-tests comparing two groups.

ML-DS, myeloid leukemia associated with Down syndrome; non-DS-AMKL, acute megakaryoblastic leukemia not associated with Down syndrome; DMSO, dimethyl sulfoxide; DAPI, 4′,6-diamidino-2-phenylindole.

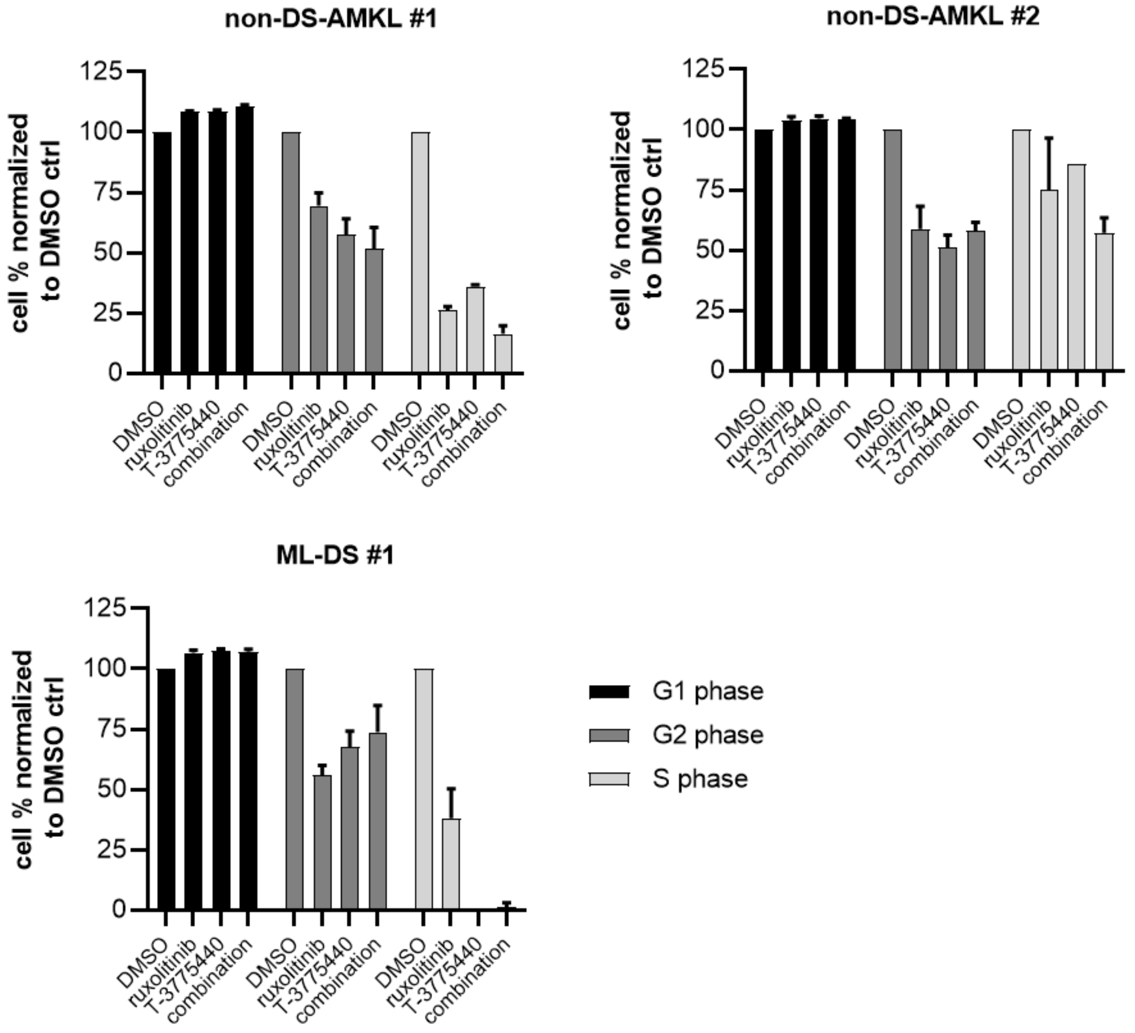

Supplemental Figure 7. LSD1 inhibitor T-3775440 blocks progression from G1 to S phase in ML-DS and non-DS-AMKL patient samples. Bar plots displaying the percentage of cells in each phase of cell cycle, as quantified via BrdU assay. All values are normalized to the corresponding DMSO control.

ML-DS, myeloid leukemia associated with Down syndrome; non-DS-AMKL, acute megakaryoblastic leukemia not associated with Down syndrome; BrdU, Bromodeoxyuridine; DMSO, dimethyl sulfoxide.

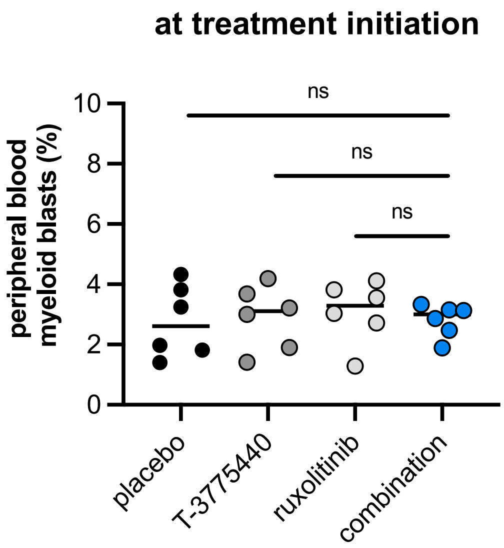

Supplemental Figure 8. Stable peripheral blood engraftment *in vivo* at the point of treatment initiation. Percentage of human myeloid blasts in the peripheral blood of humanized immunodeficient mice transplanted with the ML-DS #1 patient sample at the starting point of the treatment period. Groups treated with DMSO, T-3775440, ruxolitinib, or the combination of both drugs are shown separately. ns P>0.05; P values are derived from two-tailed Student's t-tests comparing two groups.

ML-DS, myeloid leukemia associated with Down syndrome.

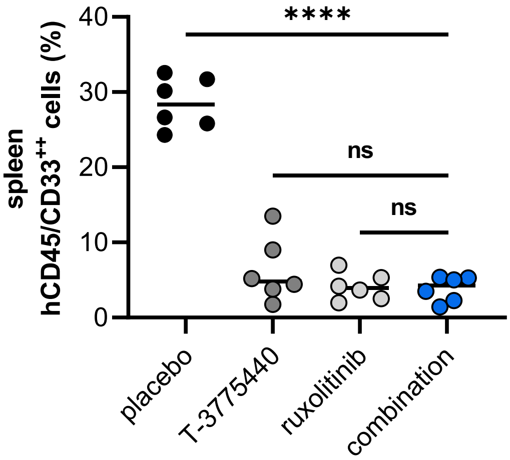

Supplemental Figure 9. LSD1 inhibitor T-3775440 or ruxolitinib monotherapy, or the combination of both drugs, all significantly reduce leukemic infiltration in the spleens of transplanted mice. Percentage of human myeloid blasts in the spleens of humanized immunodeficient mice transplanted with the ML-DS #1 patient sample, after seven days of treatment with placebo, T-3775440, ruxolitinib, or a combination of both drugs. ns $P>0.05$, ****$P<0.0001$; P values are derived from two-tailed Student's t-tests comparing two groups.
ML-DS, myeloid leukemia associated with Down syndrome.

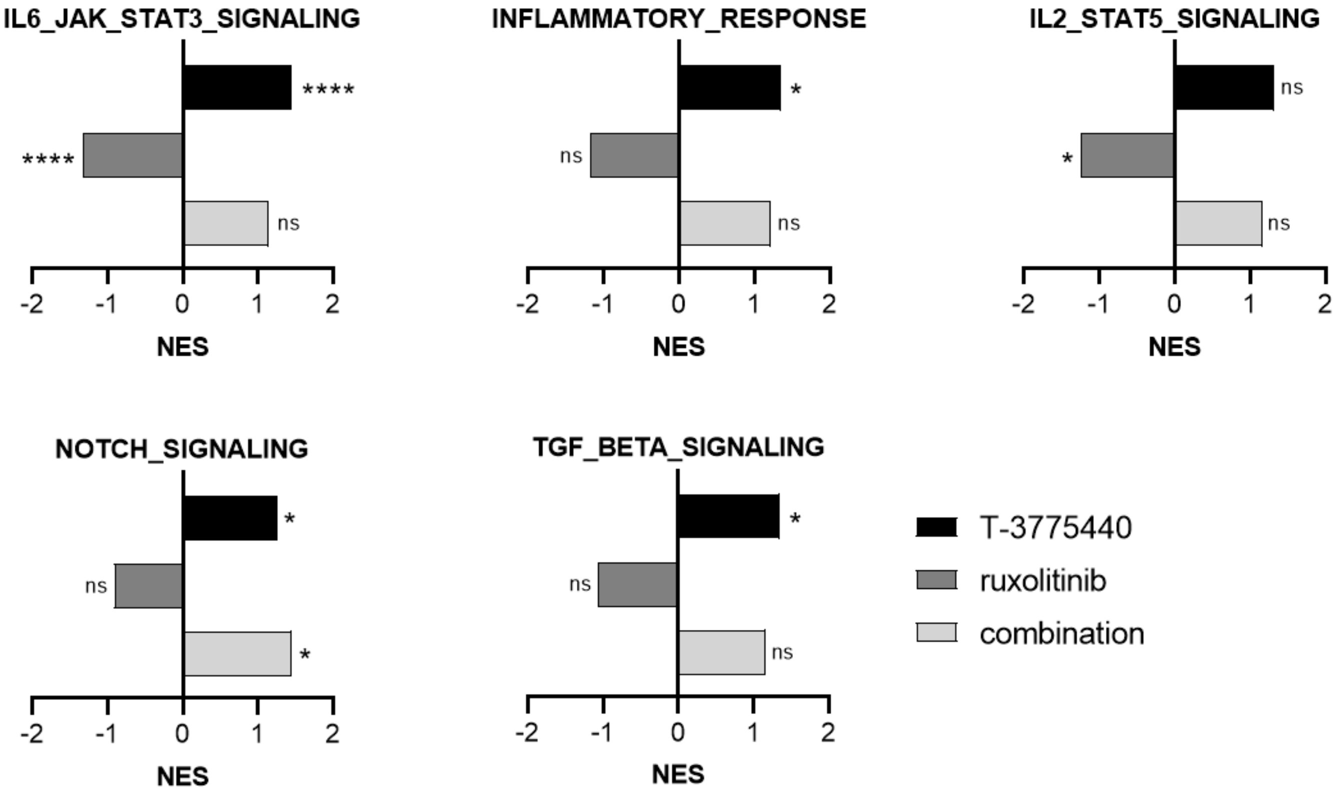

Supplemental Figure 10. GSEA reveals upregulation of cytokine signaling and pathways involved in differentiation after treatment of ML-DS patient samples with the LSD1 inhibitor T-3775440. Bar plots depicting normalized enrichment scores (NES) for the respective pathways, as determined using GSEA. This analysis was performed using RNA sequencing data from the patient samples ML-DS #1 and #2 after two days of treatment with DMSO, T-3775440, ruxolitinib, or a combination of both drugs. The DMSO treated cells were used as controls for GSEA. ns $P>0.05$, * $P<0.05$, **** $P<0.0001$; P values are calculated by the GSEA software.

GSEA, gene set enrichment analysis; ML-DS, myeloid leukemia associated with Down syndrome; NES, normalized enrichment score; DMSO, dimethyl sulfoxide.

Supplemental References

1. Ianevski A, Giri AK, Aittokallio T. SynergyFinder 2.0: visual analytics of multi-drug combination synergies. *Nucleic acids research* 2020; **48**: W488-W493.
2. Rongvaux A, Willinger T, Martinek J, Strowig T, Gearty SV, Teichmann LL, et al. Development and function of human innate immune cells in a humanized mouse model. *Nature biotechnology* 2014; **32**: 364–372.
3. Faul F, Erdfelder E, Lang A-G, Buchner A. G*Power 3: a flexible statistical power analysis program for the social, behavioral, and biomedical sciences. *Behavior research methods* 2007; **39**: 175–191.
4. Subramanian A, Tamayo P, Mootha VK, Mukherjee S, Ebert BL, Gillette MA, et al. Gene set enrichment analysis: a knowledge-based approach for interpreting genome-wide expression profiles. *Proceedings of the National Academy of Sciences of the United States of America* 2005; **102**: 15545–15550.
5. Mootha VK, Lindgren CM, Eriksson K-F, Subramanian A, Sihag S, Lehar J, et al. PGC-1alpha-responsive genes involved in oxidative phosphorylation are coordinately downregulated in human diabetes. *Nature genetics* 2003; **34**: 267–273.
6. Mi H, Muruganujan A, Casagrande JT, Thomas PD. Large-scale gene function analysis with the PANTHER classification system. *Nature protocols* 2013; **8**: 1551–1566.
7. Carbon S, Mungall C. *Gene Ontology Data Archive*. Zenodo, 2018.
8. R Core Team. R: A Language and Environment for Statistical Computing 2020. https://www.R-project.org/.
